# Long non-coding RNA CCAT1 promotes gallbladder cancer development via negative modulation of miRNA-218-5p

**DOI:** 10.1038/cddis.2014.541

**Published:** 2015-01-08

**Authors:** M-Z Ma, B-F Chu, Y Zhang, M-Z Weng, Y-Y Qin, W Gong, Z-W Quan

**Affiliations:** 1 Department of General Surgery, Xinhua Hospital, Shanghai Jiaotong University School of Medicine, Shanghai, China; 2 Department of General Surgery, Taixing People's Hospital, Yangzhou University School of Medicine, Jiangsu Province, Taixing, China; 3 Department of Gastroenterology, Yijishan Hospital Affiliated to Wannan Medical College, Wuhu, Anhui, China

## Abstract

Protein-coding genes account for only ~2% of the human genome, whereas the vast majority of transcripts are non-coding RNAs (ncRNAs) including long ncRNAs (lncRNAs). A growing volume of literature has proposed that lncRNAs are important factors in cancer. Colon cancer-associated transcript-1 (CCAT1), an lncRNA, which was first identified in colon cancer, was previously shown to promote tumor development and be a negative prognostic factor in gastric cancer. However, the mechanism through which CCAT1 exerts its oncogenic activity remains largely unknown. Recently, a novel regulatory mechanism has been proposed in which RNAs can cross-talk with each other via competing shared for microRNAs (miRNAs). The proposed competitive endogenous RNAs could mediate the bioavailability of miRNAs on their targets, thus imposing another level of posttranscriptional regulation. In this study, we demonstrated that CCAT1 was upregulated in gallbladder cancer (GBC) tissues. CCAT1 silencing downregulated, whereas CCAT1 overexpression enhanced the expression of miRNA-218-5p target gene *Bmi1* through competitively ‘spongeing' miRNA-218-5p. Our data revealed that CCAT1 knockdown impaired the proliferation and invasiveness of GBC cells, at least in part through affecting miRNA-218-5p-mediated regulation of Bmi1. Moreover, CCAT1 transcript level was correlated with Bmi1 mRNA level in GBC tissues. Together, these results suggest that CCAT1 is a driver of malignancy, which acts in part through ‘spongeing' miRNA-218-5p.

Gallbladder cancer (GBC) is the common biliary tract cancer and the fifth most common gastrointestinal malignancy.^1^ Although tremendous efforts have been put into clarifying the pathophysiological mechanisms contributing to gallbladder carcinoma, much of it remains largely unknown.^2, 3^ Therefore, it is vital to reveal the molecular mechanisms of the progression of GBC for the development of effective therapies.

The genome-sequencing projects revealed that the human genome comprises <2% protein-coding genes and >90% of the genome is transcribed as non-coding RNAs (ncRNAs).^4^ The ncRNA class now comprises a vast diversity of subclasses organized based on their size, structure, function and conservation. microRNAs (miRNAs), a class of short ncRNA molecules that range in size from 19 to 25 nucleotides (nt), have been one of the well-characterized classes of ncRNAs in GBC.^5, 6, 7^ Unlike the miRNAs, long ncRNAs (lncRNAs) are by definition >200 nt in length. A growing volume of literature has proposed that lncRNAs have an important role in cancer.^8, 9, 10^ Although much of the focus in ncRNA research (such as miRNAs and lncRNAs) has been on the regulation of protein-coding genes mediated by them, it has been suggested that ncRNAs could form a well-orchestrated regulatory interaction network: miRNAs and lncRNAs could interact with each other, imposing an additional level of posttranscriptional regulation.^11, 12, 13, 14^ An example of this type of regulation is exemplified by lncRNA GAS5, which binds miRNA-21 and forms a regulatory interaction.^12^

Colon cancer-associated transcript-1 (CCAT1), a 2628-bp lncRNA that maps to chromosome 8q24.21, was first found to be upregulated in colon cancer.^15^ Previous studies suggest that CCAT1 is upregulated in gastric carcinoma tissues and colon carcinoma tissues compared with adjacent normal tissues, and can be activated by c-Myc.^8, 15^ Moreover, abnormally expressed CCAT1 promotes cell proliferation and migration in gastric cancer cells.^8^ However, although CCAT1 has a vital role in cancer, little is known about the mechanism through which CCAT1 exerts its oncogenic activity, and the interaction between CCAT1 and miRNAs remains largely unknown.

In the present study, we demonstrated that CCAT1 was upregulated in GBC tissues. CCAT1 upregulated the miRNA-218-5p target gene *Bmi1* by competitively ‘spongeing' miRNA-218-5p and then promoted the proliferation and invasiveness of GBC cells. CCAT1 may function as a part of the ‘competitive endogenous RNA (ceRNA)' network.^16^

## Results

### CCAT1 was upregulated in GBC

**Figure 1:**
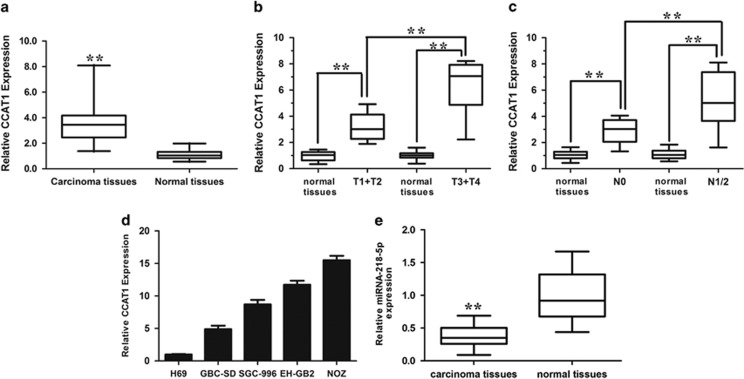
Expression levels of CCAT1 and miRNA-218-5p in GBC and its clinical significance. (**a**) Difference in expression levels of CCAT1 between GBC tissues and matched non-tumor gallbladder tissues. The expression of CCAT1 was normalized to GADPH. The statistical differences between samples were analyzed with paired samples *t*-test (*n*=40, *P*<0.0001). (**b**) Relationship between CCAT1 expression and primary tumor growth (*P*<0.0001). The expression of CCAT1 in T1+T2/T3+T4 stage tumors was normalized to correspondingly paired normal tissues. (**c**) Relationship between CCAT1 expression and lymph node metastasis (*P*<0.0001). The expression of CCAT1 in N1+2/N0 stage tumors was normalized to correspondingly paired normal tissues. The relative expression fold change of mRNAs was calculated by the 2^−ΔΔCt^ method. Horizontal lines in the box plots represent the medians, the boxes represent the interquartile range and the whiskers represent the 2.5th and 97.5th percentiles. (**d**) Expression levels of CCAT1 in four GBC cell lines (GBC-SD, SGC-996, EH-GB2 and NOZ) and a non-tumorigenic biliary epithelial cell line (H69). The expression of CCAT1 was normalized to that in H69. The statistical differences between groups were analyzed using independent samples *t*-test. Error bars represent the mean±S.D. of triplicate experiments. *P<0.05; **P<0.01. (**e**) miRNA-218-5p is downregulated in GBC tissues compared with paired adjacent normal gallbladder tissues. miRNA-218-5p mRNA expression was analyzed by real-time PCR and normalized to GADPH. The relative expression fold change of mRNAs was calculated by the 2^−ΔΔCt^ method. Horizontal lines in the box plots represent the medians, the boxes represent the interquartile range and the whiskers represent the 2.5th and 97.5th percentiles. The statistical differences between samples were analyzed with paired samples *t*-test (*n*=40, *P*<0.0001)

The relative expression level of CCAT1 was evaluated by using real-time PCR in 40 patients with GBC and paired normal tissues. CCAT1 was upregulated in GBC tissues compared with adjacent normal tissues (Figure 1a, *P*<0.001). Furthermore, 26/40 (65%) tumor tissues expressed 1.5-fold higher CCAT1 than normal tissues. CCAT1 was more highly expressed in tumors extending beyond the gallbladder (T3+T4) compared with tumors only detected in the gallbladder (T1+T2) (Figure 1b), and more highly expressed in tumors spread to lymph nodes (N1/2) compared with tumors localized only in the gallbladder (N0) (Figure 1c). In order to further understand the significance of CCAT1 overexpression in GBC, we determined the potential associations between CCAT1 expression and patients' clinicopathological features. According to the median ratio of relative CCAT1 expression (3.682) in tumor tissues, the 40 GBC patients were classified into two groups: high-CCAT1 group (*n*=13): CCAT1 expression ratio ≥median ratio; and low-CCAT1 group (*n*=27): CCAT1 expression ratio ≤median ratio. The detailed relationships between CCAT1 expression status and clinicopathological variables of 40 patients were summarized in Supplementary Table 2. It is worth noting that high CCAT1 expression was significantly correlated with tumor status (*P*=0.029), lymph node invasion (*P*=0.018) and advanced tumor node metastasis (TNM) stage (*P*=0.023). However, CCAT1 expression level was not correlated with other parameters such as patient's gender and age (Supplementary Table 2).

We used 5′- and 3′-rapid amplification of cDNA ends RACE to map the exact sequence of CCAT1 in the cell lines used in this study. The RACE results identified two isoforms of CCAT1 (Supplementary Table 3). Next, we examined the expression level of CCAT1 in a series of GBC cell lines. H69 cell line, was used as a representative of non-tumorigenic biliary epithelial cells. Among the four invasive cell lines, the CCAT1 levels were increased as compared with that in the H69 cells (Figure 1d). We selected GBC cell line NOZ for CCAT1 knockdown and cancer cell line GBC-SD for CCAT1 overexpression, as they harbored the highest and lowest expression levels of CCAT1, respectively.

### Identification of potential CCAT1-targeting miRNAs

The function of lncRNAs in human cancer may have to do with their ability to regulate protein expression.^9, 10, 17^ For example, LncRNA-Dreh could combine with the intermediate filament protein vimentin and repress its expression.^17^ A growing volume of literature has proposed that ncRNAs may participate in the ceRNAs regulatory network.^11, 12, 13, 14^ For example, miRNA-21 and GAS5 formed a reciprocal repression feedback loop.^12^ We performed a search for miRNAs that have complementary base pairing with lncRNA CCAT1, using online software program starbase v2.0 (http://starbase.sysu.edu.cn/mirLncRNA.php).^18^ The search results demonstrated that 22 miRNAs formed complementary base pairing with CCAT1 (Supplementary Table 4). We profiled the expression levels of the 22 miRNAs in GBC cell NOZ in response to CCAT1 knockdown. As demonstrated in Supplementary Table 5, there were three miRNAs that were upregulated more than 2.5-fold in response to CCAT1 inhibition. We then focused on miRNA-218-5p, which is of the greatest fold-change in response to CCAT1-specific siRNA.

The expression levels of miRNA-218-5p were also determined in GBC tissues from Figure 1. The miRNA-218-5p was markedly downregulated in GBC tissues compared with adjacent normal tissues (Figure 1e, *P*<0.001).

### CCAT1 negatively regulated the expression of miRNA-218-5p

**Figure 2:**
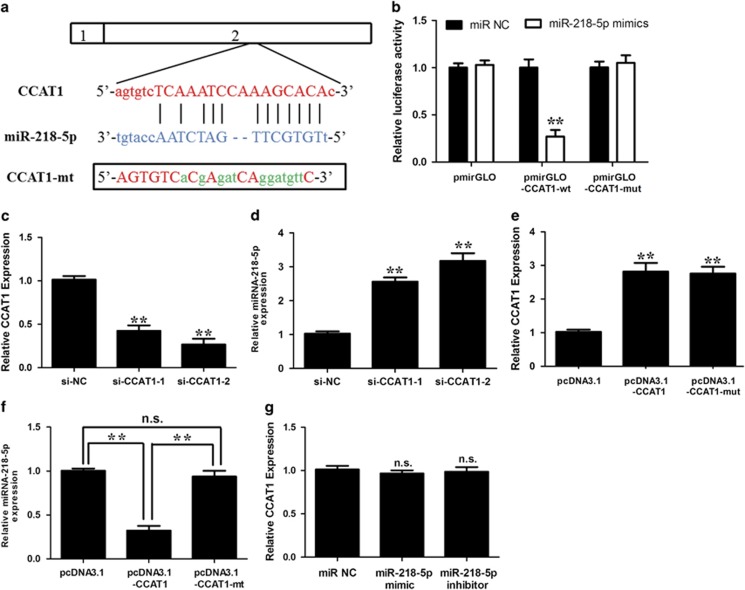
Identification of miRNA-218-5p as a target of CCAT1. (**a**) Alignment of potential CCAT1 base pairing with miRNA-218-5p as identified by Starbase v2.0 (http://starbase.sysu.edu.cn/mirLncRNA.php). CCAT1 (top) consist of two exons, where the putative binding site is in exon 2. The mutant CCAT1 at putative binding site. (**b**) Luciferase activity in NOZ cells cotransfected with miRNA-218-5p mimics and luciferase reporters containing nothing, CCAT1 or mutant transcripts. Data are presented as the relative ratio of firefly luciferase activity of Renilla luciferase activity. (**c**) CCAT1-specific siRNA 1,2 reduced the endogenous CCAT1 mRNA level in NOZ cells. (**d**) Upregulation of miRNA-218-5p by si-CCAT1. NOZ cells were transfected with control siRNA or si-CCAT1-1/2, and total RNA was isolated 48 h after transfection. (**e**) CCAT1-wt or CCAT1-mut was overexpressed in GBC-SD cells. The expression level of mutant clone is similar to that of WT overexpression clone. (**f**) This mutant CCAT1 clone revealed no significant suppression of miRNA-218-5p compared with wild-type of CCAT1. (**g**) GBC-SD cells were transfected with miRNA-218-5p mimics or inhibitor, and total RNA was isolated 48 h after transfection. Error bars represent the mean±S.D. of triplicate experiments. *P<0.05; **P<0.01; n.s., not significant

According to the prediction results, there was one putative binding site in exon 2 of CCAT1 (Figure 2a). To confirm the direct binding between CCAT1 and miRNA-218-5p, luciferase reporter constructs were generated. We observed that miRNA-218-5p mimics reduced the luciferase activities of wild-type (WT) CCAT1 reporter vector. However, luciferase activities in cells transfected with CCAT1 mutant and the miRNA-218-5p mimics were almost comparable to that of control cells (Figure 2b). These data confirmed the direct binding between CCAT1 and miRNA-218-5p.

To avoid off-target effects, we designed two CCAT1-specific siRNAs targeting different regions of CCAT1. Forty-eight hours after transfection, CCAT1 and miRNA-218-5p expression levels were measured. CCAT1 siRNAs significantly decreased the endogenous CCAT1 transcript level in NOZ cells (Figure 2c); meanwhile, CCAT1 siRNAs upregulated the expression level of miRNA-218-5p in NOZ cells (Figure 2d). In contrast, ectopic expression of CCAT1 increased the transcript level of CCAT1 in GBC-SD cells (Figure 2e), while it decreased the expression level of miRNA-218-5p (Figure 2f). We overexpressed CCAT1-wt or CCAT1-mut in GBC-SD cells (Figure 2e). The expression level of mutant clone is similar to that of WT overexpression clone. This mutant CCAT1 clone revealed no significant suppression of miRNA-218-5p compared with WT of CCAT1 (Figure 2f). However, there was no obvious difference in CCAT1 level after ectopic expression or knockdown of miRNA-218-5p (Figure 2g).

### The underlying mechanism of the negative regulation of miRNA-218-5p by CCAT1

**Figure 3:**
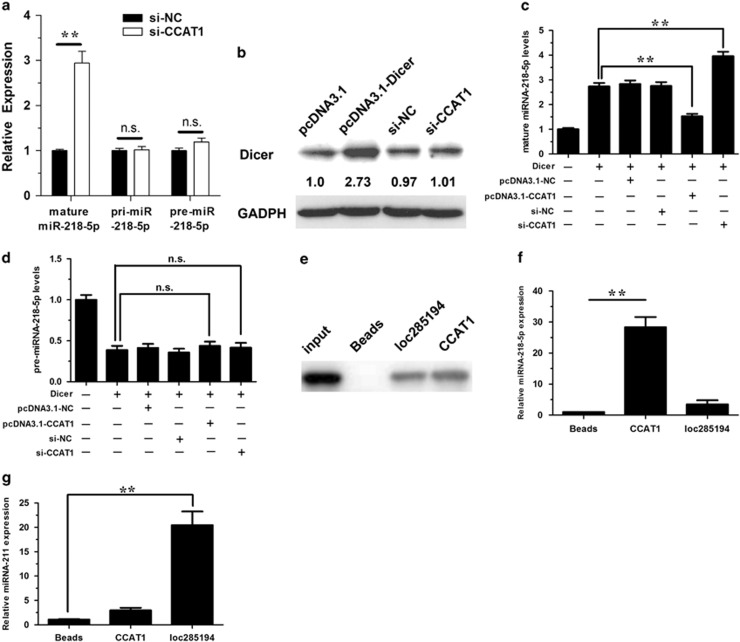
The underlying mechanism of the negative regulation of miRNA-218-5p by CCAT1. (**a**) Effect of CCAT1 on mature miRNA-218-5p, pri-miRNA-218-5p and pre-miRNA-218-5p. (**b**) NOZ cells were transfected with pcDNA3.1 or pcDNA3.1-Dicer, or si-NC or si-CCAT1 for 48 h, and western blot analysis was performed. (**c** and **d**) NOZ cells were cotransfected with pcDNA3.1-Dicer and pcDNA3.1-CCAT1 or si-CCAT1. Forty-eight hours after transfection, cells were collected. Mature miRNA-218-5p (**c**) and pre-miRNA-218-5p (**d**) were analyzed by qRT-PCR. (**e**) Pull-down of Ago2 by biotin-labeled CCAT1 or loc285194 RNA probe, as detected by western blotting. The loc285194 lane was composed from the same gel with the same contrast. Empty vector (Beads) was used as a negative control. Loc285194 was used as a positive control. (**f** and **g**) RIP followed by microRNA qRT-PCR to detect microRNAs endogenously associated with CCAT1 and loc285194. The expression level of miRNA was normalized to that in empty vector (Beads). Error bars represent the mean±S.D. of triplicate experiments. *P<0.05; **P<0.01, n.s., not significant

To explore the underlying mechanism of such a negative regulation of miR-218 by CCAT1, we examined the expression level of pri-miRNA-218-5p, pre-miRNA-218-5p and mature miRNA-218-5p in response to CCAT1 knockdown. As illustrated in Figure 3a, although CCAT1 siRNA significantly upregulated mature miRNA-218-5p, it had almost no effect on the expression levels of pri-miRNA-218-5p and pre-miRNA-218-5p. The data suggest that this negative regulation effect might be through a posttranscriptional mechanism. Mature miRNAs are generated through a two-step processing by Drosha and Dicer.^19^ The initial process occurs in the nucleus that Drosha-DGCR8 complex cleaves pri-miRNA into pre-miRNA. The second processing step occurs in the cytoplasm when pre-miRNA was cleaved by Dicer complex to create a mature miRNA duplex.^20^ To further explore whether the downregulation of mature miRNA-218-5p by CCAT1 was through the interference of the processing of Dicer, we applied Dicer to assay the levels of pre-miRNA-218-5p and mature miRNA-218-5p. Ectopic expression of Dicer significantly upregulated the protein level of Dicer (Figure 3b). What's more, CCAT1 had no significant effect on the expression level of Dicer (Figure 3b). Our results demonstrated that although Dicer overexpression increased the level of mature miRNA-218-5p (Figure 3c) and in the meantime decreased the level of pre-miRNA-218-5p (Figure 3d), CCAT1 overexpression inhibited the increase of mature miRNA-218-5p induced by Dicer, and CCAT1 silencing had an opposite effect (Figure 3c). Both CCAT1 overexpression and silencing had no significant effect on the expression level of pre-miRNA-218-5p with Dicer overexpression (Figure 3d). Taken together, it suggests that the regulation of miRNA-218-5p by CCAT1 is through another mechanism.

Previous studies revealed that miRNAs are present in the cytoplasm in the form of miRNA ribonucleoprotein complexes that also contain Ago2, the core component of the RNA-induced silencing complex (RISC). miRNAs exert their gene-silencing functions through RISC.^21^ Potential miRNA targets can be isolated from this complex after Ago2 co-immunoprecipitation,^11, 22^ as Ago2 is a vital component of RISC complex necessary for siRNA or miRNA-mediated gene silencing. Furthermore, CCAT1 is shown to be a cytoplasm-located lncRNA.^23^

We performed RNA pull-down experiments by using CCAT1 probe and then examined Ago2 and miRNA-218-5p simultaneously as described previously,^11, 12^ to determine whether CCAT1 and miRNA-218-5p are in the same RISC complex. Loc285194 (Liu *et al.*^11^) was used as a positive control. A biotin-labeled CCAT1 RNA probe was synthesized and mixed with the cellular extract. After pull-down experiment with streptavidin beads, RNA or protein that interacts with the probe is expected to co-precipitate with the biotin-labeled probe. The *in vitro* RNA pull-down experiment was performed to confirm the direct physical association between CCAT1 and Ago2. As a result, we detected Ago2 (Figure 3e). Furthermore, we detected miRNA-218-5p in the same pellet, supporting that miRNA-218-5p is *bona fide* CCAT1-targeting miRNA (Figure 3f). To demonstrate the specificity of the association between CCAT1 and miRNA-218-5p, we detected miRNA-211, which was shown to be negatively regulated by loc285194 (Liu *et al.*^11^) and did not form complementary base pairing with CCAT1 according to our prediction results. Quantitative reverse-transcription PCR (qRT-PCR) analysis revealed that loc285194 was significantly enriched for miRNA-211 compared with the empty vector (Beads) and CCAT1 (Figure 3g). Our data suggest that the regulation between miRNA-218-5p and CCAT1 might be in a way similar to the miRNA-mediated silencing of protein-coding genes.^11, 14^

### CCAT1 negatively regulates *Bmi1*, a miRNA-218-5p target gene

**Figure 4:**
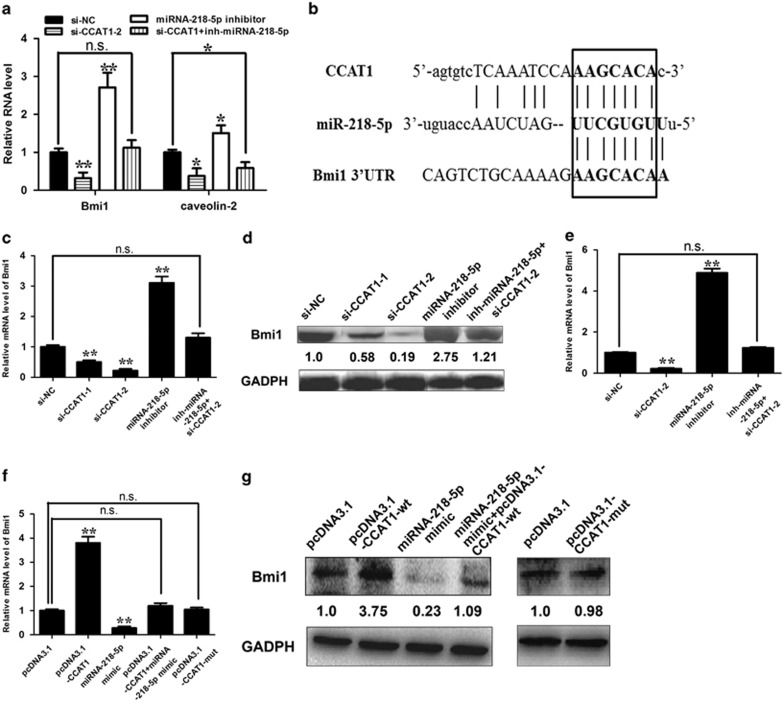
Regulation of Bmi1 by CCAT1. (**a**) NOZ cells were transfected with si-NC, si-CCAT1, si-CCAT1+miRNA-218-5p inhibitor and miRNA-218-5p inhibitor. qRT-PCR was performed 48 h post transfection. (**b**) Nucleotide resolution of miRNA-binding sites in CCAT1 and Bmi1. As indicated, CCAT1 and Bmi1 shared the same miRNA-responsive element in their sequences. miRNA-218-5p is a validated Bmi1-targeting miRNA. The mRNA (**c**) or protein (**d**) levels of Bmi1 in NOZ cells transfected with si-NC, si-CCAT1, si-CCAT1+miRNA-218-5p inhibitor and miRNA-218-5p inhibitor. (**e**) The mRNA levels of Bmi1 in GBC-SD cells transfected with si-NC, si-CCAT1-2, si-CCAT1-2+miRNA-218-5p inhibitor and miRNA-218-5p inhibitor. The mRNA (**f**) or protein (**g**) levels of Bmi1 in GBC-SD cells transfected with pcDNA3.1, pcDNA3.1-CCAT1, miRNA-218-5p mimic, pcDNA3.1-CCAT1+miRNA-218-5p mimic and pcDNA3.1-CCAT1-mut. Error bars represent the mean±S.D. of triplicate experiments. *P<0.05; **P<0.01

Having demonstrated that CCAT1 could negatively regulate miRNA-218-5p expression, we then examined the functional aspect. miRNA-218-5p was shown to be downregulated in a variety of carcinomas, including mesenchymal glioblastoma,^24^ pancreatic cancer^25^ and renal cell carcinoma,^26^ and exhibits tumor-suppressive activities,^24, 25, 26, 27^ while CCAT1 was shown to promote tumor progression.^8^ In the first place, we examined whether CCAT1 has any effect on miRNA-218-5p target genes and found that CCAT1 knockdown inhibited the expression of Bmi1 (Tu *et al.*^27^) and caveolin-2 (Yamasaki *et al.*^26^), whereas co-transfection of miRNA-218-5p inhibitor attenuated this inhibition (Figure 4a). The incomplete rescue of caveolin-2 by miRNA-218-5p inhibitor suggests that other mechanisms might also be involved in its regulation. Previous studies suggest that miRNA-218-5p inhibits tumor invasion, migration and proliferation by targeting the polycomb group gene *Bmi1* (Tu *et al.*^27^). As CCAT1 shares regulatory miRNA-218-5p with Bmi1 (Figure 4b), we would like to explore whether CCAT1 could modulate miRNA-218-5p and then Bmi1. We observed that although si-CCAT1 decreased Bmi1 transcript and protein levels, miRNA-218-5p inhibitor abrogated this decrease in NOZ cells (Figures 4c and d). Similar phenomenon was observed in GBC-SD cells (Figure 4e). In GBC-SD cells, although ectopic expression of CCAT1 upregulated Bmi1 at both transcript and protein levels, miRNA-218-5p mimic relieved the increase (Figures 4f and g). Yet, CCAT1-mut had no significant effect on the expression of Bmi1 (Figure 4g).

**Figure 5:**
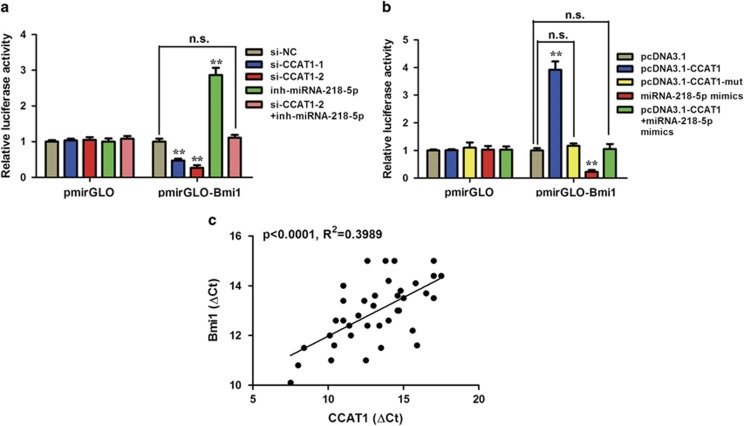
Regulation of Bmi1 by CCAT1 depends on the regulation of the 3′-UTR of Bmi1. (**a** and **b**) Luciferase activity in NOZ cells (**a**) transfected with luciferase reporters containing Bmi1 3′-UTR or nothing. Data are represented as the relative ratio of firefly luciferase activity to Renilla luciferase activity. Error bars represent the mean±S.D. of triplicate experiments. *P<0.05; **P<0.01; n.s., not significant. (**c**) The correlation between CCAT1 transcript level and Bmi1 mRNA level was measured in 40 gallbladder tissues. The ΔCt values (normalized to GADPH) were subjected to Pearson's correlation analysis

Our results revealed that CCAT1 and Bmi1 shared the same miRNA-responsive element in their sequences and displayed the same miRNA-218-5p-dependent regulation pattern. To explore whether the observed effects depends on the regulation of the 3′ untranslated regions (3′-UTR) of Bmi1, a luciferase reporter plasmid containing the 3′-UTR of Bmi1 was constructed. Luciferase plasmid was transfected into NOZ and GBC-SD cells. As illustrated in Figure 5a, CCAT1 knockdown decreased the luciferase activity in the Luc–Bmi1-3′-UTR-transfected NOZ cells, which was rescued by miRNA-218-5p inhibitor. On the other hand, ectopic expression of CCAT1 WT, but not CCAT1-mut, increased the luciferase activity in the Luc–Bmi1-3′-UTR-transfected GBC-SD cells. However, miRNA-218-5p mimics abolished this upregulation (Figure 5b). Furthermore, CCAT1 transcript level was significantly correlated with miRNA-218-5p mRNA level in GBC tissues (Figure 5c).

### CCAT1's oncogenic activity is in part through negative regulation of miRNA-218-5p and then modulating Bmi1 *in vitro*

**Figure 6:**
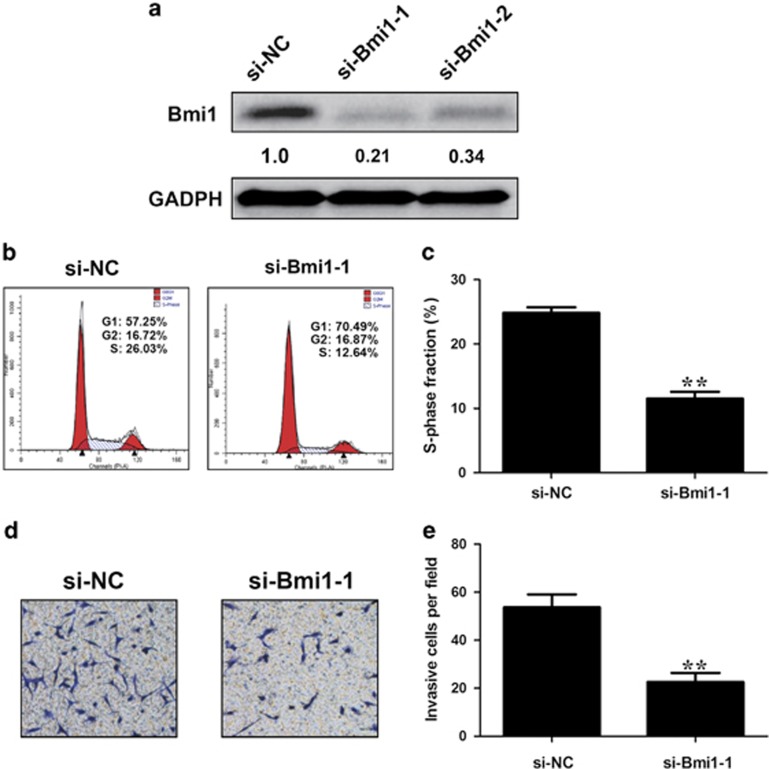
Bmi1 promotes the growth and invasion of GBC cells. (**a**) Bmi1-specific siRNA 1,2 effectively suppressed the protein level of Bmi1. (**b**, **c**) Flow cytometric analysis was performed in NOZ cells. Data were expressed as percentage distribution of cells in G0/G1, S and G2/M phases of the cell cycle. (**d**, **e**) The invasive ability of NOZ cells can be blocked by Bmi1 downregulation. Error bars represent the mean±S.D. of triplicate experiments. *P<0.05; **P<0.01

To investigate the biological association between CCAT1 and miRNA-218-5p, we employed gain- and loss-of-function approaches. Bmi1 siRNAs resulted in significant downregulation of the protein level of Bmi1 (Figure 6a). The percentage of S-phase cells and the number of invasive cells were reduced after Bmi1 knockdown (Figures 6b–e). The data suggest that Bmi1 has an important role in proliferation and migration of GBC.

**Figure 7:**
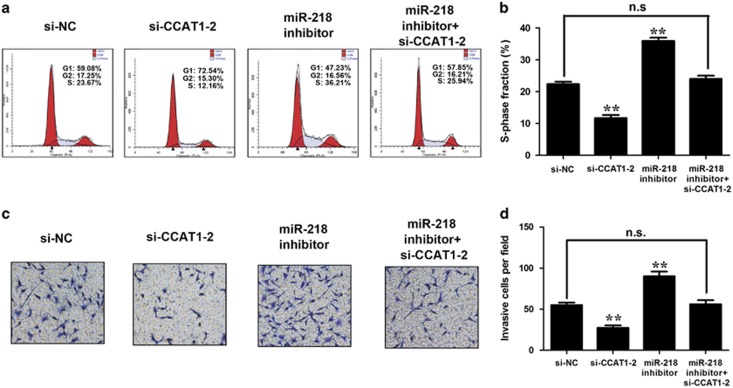
CCAT1's oncogenic activity is in part through negative regulation of miR-218 and then modulating Bmi1. (**a**) Flow cytometric analysis was performed. Data were expressed as percentage distribution of cells in G0/G1, S and G2/M phases of the cell cycle. (**b**) Si-CCAT1 induced a reduction of S-phase fraction cells in NOZ cells, which can be rescued by miRNA-218 inhibitor. miRNA-218 inhibitor alone increased the percentage of S-phase cells. (**c**) Forty-eight hours after transfection, transwell invasion assay was performed. (**d**) The invasive ability of NOZ cells can be blocked by CCAT1 downregulation. The si-CCAT1-blocked invasive ability of NOZ cells was rescued by miRNA-218 inhibitor, and miRNA-218 inhibitor alone increased the invasive ability. Error bars represent the mean±S.D. of triplicate experiments. *P<0.05; **P<0.01; n.s., not significant

A decrease in the percentage of S-phase cells was observed in GBC cell NOZ after transfection of CCAT1 siRNA but was reversed by cotransfection of miRNA-218-5p inhibitor (Figures 7a and b). Similar phenomenon was observed in invasion (Figures 7c and d) of GBC cells. All these data imply that the oncogenic activity of CCAT1 is partly associated with the regulation of miRNA-218-5p and then Bmi1.

### CCAT1's oncogenic activity is in part through negative regulation of miRNA-218-5p *in vivo*

**Figure 8:**
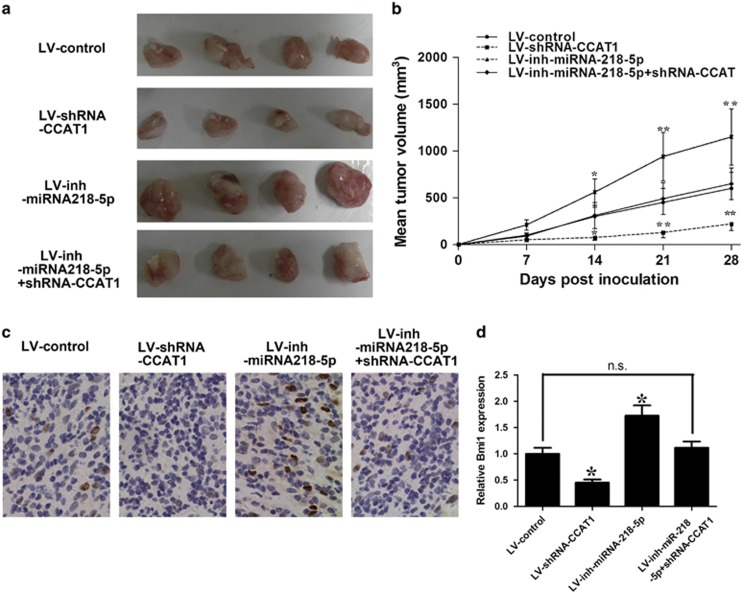
CCAT1's oncogenic activity is in part through negative regulation of miRNA-218-5p *in vivo*. CCAT1 knockdown suppressed tumor growth in subcutaneous implantation mouse models of NOZ cells and miRNA-218-5p inhibitor abrogated this suppression in tumor growth. Tumor volumes (**a**) and tumor growth curves (**b**) of subcutaneous implantation models of GBC are shown. (**c**, **d**) Immunohistochemical staining of Bmi1 demonstrated that CCAT1 silencing inhibited the aggressive phenotype of gallbladder cells *in vivo*, as indicated by the expression of Bmi1-positive cells and miRNA-218-5p inhibitor abolished the inhibition.*P<0.05, **P<0.01

To provide additional evidence to the idea that CCAT1's oncogenic activity is in part through the negative regulation of miRNA-218-5p, we stably transfected NOZ cells with a lentivirus construct containing desired vector. The cells were then injected subcutaneously into nude mice. Tumor volume was measured on a weekly basis and mice were killed 4 weeks after cancer cell inoculation. Palpable tumor formed within 5 days. After 4 weeks, we observed a decrease in tumor growth in the NOZ-shRNA-CCAT1 group compared with the NOZ-vector group and miRNA-218-5p inhibitor abrogated this decrease in tumor growth (Figures 8a and b). Furthermore, our data demonstrated that the expression level of Bmi1 of the tumor from the shRNA-CCAT1 group was lower compared with the control group with immunochemistry analysis and miRNA-218-5p inhibitor abrogated the decrease (Figures 8c and d)

## Discussion

Recent evidence has shown that ncRNAs have an important role in cancer pathogenesis and could provide new insights into the biology of this disease.^5, 6, 7, 8, 9, 10, 11, 12^ Over the past decade, the researches of miRNAs have dominated the field of ncRNA regulation.^5, 6, 7, 8^ However, the role of lncRNAs in the tumorigenesis of GBC remains largely unknown. Understanding the precise molecular mechanism by which lncRNAs function would facilitate the development of lncRNA-directed diagnostics and therapeutics against cancers. In this study, we provide evidence that CCAT1 exhibits oncogenic activities partly through modulation of miRNA-218-5p and then Bmi1.

A growing number of reports suggests the existence of a widespread interaction network involving ceRNAs, where ncRNAs could regulate modulatory RNA by binding and titrating them off their binding sites on protein coding messengers.^13, 28^ An example of this type of regulation is exemplified by HULC, an lncRNA highly upregulated in liver cancer, whose upregulated expression is in part to its inhibitory effects on the expression and activity of miRNA-372 (Wang *et al.*^29^). Similar report suggests that H19 acts as a molecular sponge to modulate let-7 availability and inhibits muscle differentiation through Antagonizing let-7 (Kallen *et al.*^30^). Han *et al.*^31^ revealed that miRNA-125b suppresses the expression of oncogenic lncRNA MALAT1. Liu *et al.*^11^ demonstrated a reciprocal repression between loc285194 and miRNA-211 in colon cancer. Similar phenomenon was observed between GAS5 and miRNA-21 (Zhang *et al.*^12^). The entire spectrum of ncRNA regulatory layer, especially the interaction between lncRNA–miRNA remains to be explored.

CCAT1 is an lncRNA initially characterized in colon cancer.^15^ CCAT1 was found to be activated by c-Myc, and is crucial for cancer cell invasion and proliferation in gastric cancer.^8^ However, the mechanism through which CCAT1 exerts its oncogenic functions remains to be explored. In this study, we would like to explore the interaction between CCAT1 and miRNAs in GBC. We first examined the expression pattern of CCAT1 in GBC tissues. CCAT1 was found to be upregulated in GBC tissues compared with adjacent normal tissues and its expression pattern was associated with tumor status and lymph node status. It is worth mentioning that although we did make efforts to collect the normal epithelial tissues as the adjacent normal tissues, as it is the normal epithelial layer in the gallbladder that gives rise to the carcinoma, it is inevitable that the ‘normal' samples contain some non-epithelial tissue. Thus, it might bring some deviance to the expression levels of CCAT1 in the adjacent normal tissues. However, we performed two quality controls over the selection of adjacent normal tissues to minimize the deviances.

We performed a search for miRNAs that had complementary base pairing with lncRNA CCAT1 and identified 22 miRNAs that formed complementary base pairing with CCAT1. The expression changes of the 22 miRNAs in response to CCAT1 knockdown were examined. We focused on miRNA-218-5p, as it is of the greatest fold change. Knockdown of CCAT1 increased the expression of miRNA-218-5p, while ectopic expression of CCAT1 induced the downregulation of miRNA-218-5p and the miRNA-218-5p-binding site is indispensable for the CCAT1-mediated repression.

Recent studies suggest that lncRNAs may exert functions through targeting miRNAs. H19 was shown to inhibit muscle differentiation through Antagonizing let-7 (Kallen *et al.*^30^). linc-MD1 was demonstrated to ‘sponge' miR-133 and miR-133 to regulate muscle differentiation.^32^ A recently identified lncRNA, CHRF, was demonstrated to regulate cardiac hypertrophy by targeting miRNA-489 (Wang *et al.*^33^). miRNA-218-5p was demonstrated to suppress tumor growth,^24, 25, 26, 27^ while CCAT1 was shown to promote tumor progression.^8^ We studied the biological aspect of CCAT1 and miRNA-218-5p in GBC cells. The present study revealed that although knockdown of CCAT1 inhibited the proliferation and migration of GBC cells, miRNA-218-5p inhibitor reversed the effects that CCAT1-specific siRNA exerted partially through modulating Bmi1, both *in vivo* and *in vitro*. Our study suggests that CCAT1 may promote tumor development through ‘spongeing' miRNA-218-5p.

What's more, CCAT1 transcript level was found to be correlated with Bmi1 mRNA level in GBC tissues, providing additional evidence to such a regulatory network. Furthermore, we explored the underlying mechanism of the negative regulation of miRNA-218-5p by CCAT1. Our data demonstrated that CCAT1 had no effect on the expression level of pri-miRNA-218-5p and pre-miRNA-218-5p. CCAT1 inhibited the increase of mature miRNA-218-5p induced by Dicer overexpression and CCAT1 silencing had an opposite effect. Furthermore, CCAT1 had no significant effect on the expression level of Dicer. CCAT1 overexpression and silencing had no significant effect on the expression level of pre-miRNA-218-5p. It suggests this regulatory effect might be through another mechanism. We found that CCAT1 and miRNA-218-5p bound to the same RISC complex. With respect to the regulation of miRNA-218-5p by CCAT1, we hypothesize that CCAT1 promotes the degradation of miRNA-218-5p via binding to the RISC complex, in which case CCAT1 functions as an ‘miRNA,' while miRNA-218-5p acts as the ‘miRNA targeting mRNA'. The precise mechanism requires further investigation in the future.

However, the alignment between the CCAT1 and miRNA-218-5p is not very specific, as 22 miRNAs were predicted to form complementary base pairing with CCAT1. What's more, miRNA-218-5p may also act independently of lncRNA-CCAT1, as it shares homology with a number of protein-coding genes such as caveolin-2 (Yamasai *et al.*^26^) and Bmi1 (Tu *et al.*^27^).

Our study suggests another layer of regulation involving ncRNAs in both molecular and biological aspects. A better understanding of the ncRNA interaction regulatory network would definitely advance the research in tumorigenesis of GBC.

## Experimental Procedure

### Patient samples

The experiments were undertaken with the understanding and written consent of each subject. The study methodologies conformed to the standard set by the Declaration of Helsinki. This study methodology was approved by the Human Ethics Committee of Xinhua Hospital at Shanghai Jiaotong University (Shanghai, China).

The 40 gallbladder carcinoma tissues and their pair-matched adjacent normal gallbladder tissues in this study (collected postoperatively from April 2007 to May 2009) were obtained from patients who underwent radical resections at Xinhua Hospital (Shanghai Jiaotong University School of Medicine, Shanghai, China). Each sample was snap-frozen in liquid nitrogen and stored at −80 °C before RNA isolation and qRT-PCR analysis. On removal of the surgical specimen, research personnel transferred the tissues to the surgical lab immediately. Pathology faculty performed a gross evaluation of the specimen and selected the gallbladder tissues that appeared to be cancerous and tissues that appeared to be normal epithelial tissues. Next, a second level of quality control was performed on adjacent normal tissues. Histological slides were prepared from the section of frozen tissue that was selected as normal tissues. These slides were examined by experienced pathologists to determine whether the benign tissues contained any GBC cells. Benign tissues that contained residual cancer tissues were not included in this study. All patients recruited to this study did not receive any pre-operative treatments. GBC patients were staged according to the TNM staging system (the seventh edition) of the American Joint Committee on Cancer staging system. The data do not contain any information that could identify the patients. Complete clinicopathological follow-up data of the GBC patients from which the specimens were collected were available.

### Cell culture

Four human GBC cell lines (GBC-SD, SGC-996, NOZ and EH-GB2) were used in this study. GBC-SD and SGC-996 were purchased from Cell Bank of the Chinese Academy of Science (Shanghai, China). NOZ was purchased from the Health Science Research Resources Bank (Osaka, Japan). EH-GB2 was a generous gift from Eastern Hepatobiliary Surgical Hospital and Institute, The Second Military University, Shanghai, China.^34^ The non-tumorigenic human intrahepatic biliary epithelial cell line H69 was purchased from the Health Science Research Resources Bank. The cell lines were cultured in Dulbecco's modified Eagle's medium (Gibco BRL, Grand Island, NY, USA), containg 10% fetal bovine serum (FBS, HyClone, Invitrogen, Camarillo, CA, USA), as well as 100 U/ml penicillin and 100 *μ*g/ml streptomycin (Invitrogen, Carlsbad, CA, USA). Cells were maintained in a humidified incubator at 37 °C in the presence of 5% CO_2_. All cell lines have been passaged for fewer than 6 months.

### RNA preparation, RT and qPCR

Total RNA from tissues and cells was extracted using Trizol reagent (Invitrogen). RNA was reversed transcribed into cDNAs using the Primer-Script one step RT-PCR kit (TaKaRa, Dalian, China). The cDNA template was amplified by real-time RT-PCR using the SYBR Premix Dimmer Eraser kit (TaKaRa). Gene expression in each sample was normalized to GADPH expression. The primer sequences used were as follows: for GAPDH, 5′-GTCAACGGATTTGGTCTGTATT-3′ (forward) and 5′-AGTCTTCTGGGTGGCAGTGAT-3′ (reverse); for CCAT1, 5′-TTTATGCTTGAGCCTTGA-3′ (forward) and 5′-CTTGCCTGAAATACTTGC-3′ (reverse). Primers used in this study were supplied in the Supplementary Materials. Real-time RT-PCR reactions were performed by the ABI7500 system (Applied Biosystems, Carlsbad, CA, USA). The real-time PCRs were performed in triplicate. The relative expression fold change of mRNAs was calculated by the 2^−ΔΔCt^ method. Primers used in this study are listed in Supplementary Table 1.

### 5′- And 3′-rapid amplification of cDNA ends

We used the 5′- and 3′-RACE analyses to determine the transcriptional initiation and termination site of CCAT1 using a SMARTer RACE cDNA Amplification Kit (Clontech, Palo Alto, CA, USA), according to the manufacturer's instructions. The gene-specific primers used for the PCR of the RACE analysis were given at Supplementary Table 1.

### Real-time quantitative PCR of mature miRNAs

Primers were designed on the basis of miRNA mature sequence. Total RNA from cells was extracted using Trizol reagent (Invitrogen). RNA was reverse transcribed into cDNAs using PrimeScript miRNA cDNA Synthesis Kit (TakaRa), according to the manufacturer's instructions. The poly (A) was added to the 3′-end of miRNAs. A primer consisting of an oligo(dT) sequence is used for reverse transcription. The qRT-PCR reaction was conducted under the following conditions: single cycle of 300 s at 95 °C, 40 cycles of 30 s at 95 °C, followed by 20 s at 60 °C and 15 s at 72 °C. Small nuclear RNA U6 was used as internal control. The real-time PCRs were performed in triplicate. The relative expression fold change of mRNAs was calculated by the 2^−ΔΔCt^ method.

### Cell transfection

Hsa-miRNA-218-5p mimic/negative control mimic and hsa-miRNA-218-5p inhibitor/negative control inhibitor were purchased from Genechem, Shanghai, China. The siRNAs specifically targeting CCAT1 were synthesized by Invitrogen. The siRNA sequences for CCAT1 were si-CCAT1-1, 5′-CGGCAGGCATTAGAGATGAACAGCA-3′ and si-CCAT1-2, 5′-CCATTCCATTCATTTCTCTTTCCTA-3′. The siRNAs specifically targeting Bmi1 were synthesized by Invitrogen. The siRNA sequences for Bmi1 were si-Bmi1-1, 5′-GGAGGAACCTTTAAAGGAT-3′ and si-Bmi1-2, 5′-CCAGAGAGATGGACTGACA-3′. Transfections were performed using the Lipofectamine 2000 kit (Invitrogen) according to the manufacturer's instructions.

The cDNA encoding lncRNA-CCAT1 was PCR-amplified by the Pfu Ultra II Fusion HS DNA Polymerase (Stratagene, Agilent Technologies, Santa Clara, CA, USA) and subcloned into the *Hind*III and *Eco*RI sites of pcDNA3.1 vector (Invitrogen), named pcDNA3.1-CCAT1. The pcDNA3.1-CCAT1 with point mutations in the miRNA-218-5p response elements (seed sequence binding fragment 5′-TCAAATCCAAAGCACA-3′ changed to 5′-ACGAGATCAGGATGTT-3′) was synthesized using a QuikChange Site-Directed Mutagenesis kit (Stratagene) and named pcDNA3.1-CCAT1-mut (miRNA-218-5p).

The 815-nt region at the 3′-end of either CCAT1 or CCAT1-mut (miRNA-218-5p) was amplified using PCR and subcloned into the pmirGLO vector (Promega, Madison, WI, USA) for Luciferase reporter assay using the one-step directed cloning kit (Novoprotein, Shanghai, China). The 3′-UTR of Bmi1 mRNA containing the intact miRNA-218-5p family recognition sequences were PCR-amplified and subcloned into the *Sac*I and *Sal*I sites of pmirGLO vector. Primers used in this study are listed in Supplementary Table 1.

Cells were grown on six-well plates to 60% confluency and transfected using Lipofectamine 2000 (Invitrogen), according to the manufacturer's instructions. Forty-eight hours after transfection, cells were collected for qRT-PCR or western blot analyses. The final concentrations of miRNAs/plasmids employed in this study were as the followings: Bmi1 siRNA/negative control siRNA 20 nM/ml, CCAT1 siRNA/negative control siRNA 20 nM/ml, CCAT1-wt/CCAT1-mut 50 nM/ml, miRNA-218-5p mimic/negative control mimic 100 nM/ml and miRNA-218-5p inhibitor/negative control inhibitor 200 nM/ml.

### Luciferase reporter assay

pmirGLO, pmirGLO-CCAT1 wt or pmirGLO-CCAT1-mut (miRNA-218-5p) was cotransfected with miRNA-218-5p mimics or pmiRNA NC into NOZ cells by Lipofectamine-mediated gene transfer. pmirGLO or pmirGLO-Bmi1 was transfected into different NOZ cell clones and GBC-SD cell clones by Lipofectamine-mediated gene transfer. The relative luciferase activity was normalized to Renilla luciferase activity 48 h after transfection.

### RNA pull-down assay

To determine whether CCAT1 is associated with the RISC complex, we performed RNA pull-down assay using synthesized biotin-labeled CCAT1 as a probe and then detected Ago2 from the pellet by western blotting or detected miRNA-218-5p by qRT-PCR.

RNA pull-down were performed as described previously.^11^ Briefly, the DNA fragment covering has-miRNA-218-5p seed region binding site of CCAT1 was PCR-amplified using a T7-containing primer and then cloned into pCR8 (Invitrogen). In addition, lncRNA loc285194 (Liu *et al.*^11^) was also cloned and used in RNA pull-down assay as a positive control. The resultant plasmid DNA was linearized with restriction enzyme *Not*I. Biotin-labeled RNAs were *in vitro* transcribed with the Biotin RNA Labeling Mix (Roche Diagnostics, Indianapolis, IN, USA) and T7 RNA polymerase (Roche, Basel, Switzerland), treated with RNase-free DNase I (Roche) and purified with the RNeasy Mini Kit (Qiagen, Inc., Valencia, CA, USA). Cell extract (2 *μ*g) was mixed with biotinylated RNA (100 pmol). Washed Streptavidin agarose beads (100 ml) were added to each binding reaction and further incubated at room temperature for 1 h. Beads were washed briefly three times and boiled in SDS buffer, and the retrieved protein was detected by standard western blot technique.

The Ago2 antibodies used for RIP are purchased from Abcam (Cambridge, MA, USA). The co-precipitated RNAs were detected by RT-PCR. Total RNAs and controls were also assayed to demonstrate that the detected signals were from RNAs specifically binding to Ago2.

### Western blotting

Western blot analysis to assess Bmi1 and GADPH expression was carried out as described previously.^10^ GADPH primary antibodies were purchased from Sigma (St. Louis, MO, USA).

### Flow cytometric analysis

Cells transfected with desired plasmid or negative control were plated in six-well plates. After 48 h incubation, the cultures were incubated with propidium iodide for 30 min in the dark. Cultures were collected and analysed for cell cycle using a flow cytometer (FACSCalibur, BD Biosciences, San Jose, CA, USA) after propidium iodide staining. Data were expressed as percentage distribution of cells in G0/G1, S and G2/M phases of the cell cycle.

### Cell invasion assay

For the invasion assays, 48 h after transfection, 2 × 10^4^ cells in serum-free media were placed into the upper chamber of an insert (8.0 *μ*m, Millipore, Temecula, MA, USA). The chamber was precoated with Matrigel (Sigma). The chambers were then incubated for 24 h in culture medium with 10% FBS in the bottom chambers before examination. The cells on the upper surface were scraped and washed away, whereas the invaded cells on the lower surface were fixed and stained for 2 h. Finally, invaded cells were counted under a microscope and the relative number was calculated. Experiments were independently repeated in triplicate.

### Xenograft mouse model

NOZ cells (1 × 10^6^) stably expressing control shRNA or CCAT1 shRNA, or miRNA-218-5p inhibitor or CCAT1 shRNA+miRNA-218-5p inhibitor were subcutaneously injected into either side of flank area of 4-week-old female athymic nude mice (*n*=3 mice per group). Tumor volumes were measured (0.5 × length × width^2^) in mice on a weekly basis. After 4 weeks, mice were killed, and tumors were exercised and subjected to immunohistochemical analysis of Bmi1. All animal experiments were performed in animal laboratory center of Xinhua Hospital and in accordance with the Guide for the Care and Use of Laboratory Animals published by the US National Institutes of Health (NIH publication number 85-23, revised 1996). The study protocol was approved by the Animal Care and Use committee of Xinhua Hospital (approval ID: 2014041).

### Statistics

All statistical analyses were performed using SPSS 17.0 (SPSS, Chicago, IL, USA). The expression differences between GBC and matched normal tissues were analyzed using paired samples *t*-test. Pearson's coefficient correlation was used for expression correlation assay. The expression differences between high/low stages, the expression changes after transfection, S-phase fraction and invasion assay were analyzed using independent samples *t*-test. *P*-values were two-sided and a value of <0.05 was considered to be statistically significant.

## Abbreviations

ceRNA: competitive endogenous RNA
GBC: gallbladder cancer
ncRNA: non-coding RNA
miRNA: microRNA
nt: nucleotide
CCAT1: colon cancer-associated transcript-1
FBS: fetal bovine serum
RISC: RNA-induced silencing complex
