# Supplementary material for: Long non-coding RNA CCAT1 promotes gallbladder cancer development via negative modulation of miRNA-218-5p
Source: Cell Death Dis. 2015 Jan 8;6(1):e1583–. doi: 10.1038/cddis.2014.541 (PMC4669740; doi:10.1038/cddis.2014.541)
Supplement: Supplementary Table 3 [file cddis2014541x3.doc]

CCAT1 cDNA sequence.

**Isoform 1 (Main)**

TTTAAATCAT ACCAATTGAA CCGAGCCTTG TAGAAACACT ATCACCTACG CATACCTCTG CTTCTTTTCA TTAACCTGCT ATCCTCTTTA CAAATGGGAT TCTTCACCCA CTCCCTTCTT CTAGATTAGC AATGCCCTGT TAAGTAAACG AACACGAAAT TCAAAGGGAA ACAGGAGCAA TCATCATTAC CAGCTGCCGT GTTAAGCATT GCGAAAACGC TCACGATTCA CAGAAAAATC CATGCTGTTC TTTGAAGGCA TTCAAGCCTT AATAGCTAGC TGGATGAATG TTTAACTTCT AGGCCAGGCA CTACTCTGTC CCAACAATAA GCCCTGTACA TTGGGAAAGG TGCCGAGACA TGAACTTTGG TCTTCTCTGC AATCCATCTG GAGCATTCAC TGACAACATC GACTTTGAAG TTGCACTGAC CTGGCCAGCC CTGCCACTTA CCAGGTTGGC TCTGTATGGC TAAGCGTTTT CTCCTAAAAT CCCTTGAAAA CTGTGAGAAG ACCATAAGAA GATCATATCT TTAATTCTAT TTCACAAGTC ACACAATATT CCAATCAAAT ACAGATGGTT GAGAAAAGTC ATCCATCTTC CCTCCCCACC CTCCCACAGC CCCTCAACCA CTGCCCTGAA ACTTATATGC TGTTATCCGC AGCTCCATCT GGAGCATCAC AGCTACTGTC AACCCTGACG CTCTTTCTGA AAAAACACCG GATGGACATC AGAACTATTT CTTTAAGGAT GTTACTGAGC CACACAGGAA AACTTGCCTT ATGATTTTGA ATGCACGGAT CTGATTTGAC TAAACATGAT AACTAGAGAA TCACCCAATC TACTCCCATT TTCAACTCTA AATCATCAGA GTGTCTCAAA TCCAAAGCAC ACACAGACCA GCCTGGCCAA CACGGTGAAA CTCCACCCCT ACTAAAAGTA TAAAAATTAT CCAGGTGTGG TGGCGGGCGC CTGTAATCCA AGCTACTTGG GAGTCTGGAG GCAGGAGAAT CCCTTGAACC TGGGAGATGG AGGTTGCAGT GAGCAGAGAT CACACCACCG CACTCTAGCC TGGGCCACAA ATCAACAACA ACAACAACAA CAAAAAACAA AGCGCACACA GAGACTGAGG TCCTCTTTGG CATTGAGAAG ATGGCTATGC AAGTCCCAAC TAGCAAGTGC AAACTTCCCA GCTTCACTTC TGCCAGTGTC CCTTCACCCC TTCTCAACCC CACTGGGAGG CAGGAGGGTG CTTGACAATA ACAGCCTTGG CATCACTCTG CCAGGGTGTA ATAGGAACTG TTACAATTCT GAGATTCTGT GTAAGCACTG GCCTTTCTGC CTAGAATGCC TTCTCCTCTC TTTTTTAACT GCATGCTCCT ATTTATCTTT CAAAGCCCGG AAAAAATAAC ACTGCACACG GGAAATGCTC CCTTCCTACT GCAGTCATTT AGATGACTCT ATGCCATTCC ATTCATTTCT CTTTCCTACC ACAGAAGTGC TTTGAGATTT TGGAGTCAGA CTGCTTGAAC TTGAATCCTG GCCCTCTCAT CAGAGACTTG ACTTATTTTA GGCAAGTTAT ATAACCAATT TTACCTCAGT TCCTTACCCA TAAAATGGGT CTAATGAGAG TACCTACCAC ACAGAATTTT GATGAAAACT GAATGAGATG AAGGCCTTTA AGGCAGTGGT CCCCAACCCT GGGGACACAG ACAGGTACCA TTTTGTGGCC TGTTAGGAAC TGGGCCACAC AGCAGGAGGT GAGCAGTGGG TGAGTGAGAT CAGCGTTATT TACAGCTGCT CCCCATTGCT CACCTTACTG CCTGAGCTCC ACCTCCTGTC AGATCAGCAG TGGCATTAAA TTCTCATAGC AGCACAAACC CTGTCATGAA CTGCACATGC GAGGGATCTA GGTTGTGCGC TCCTTATGAG AATCTAATGC CTAATGACCT GTCACCGTCT CCCATCACCC CTAGATGGGA GTGTCTAGTT GCAGGAAACA AGCTCAGGGC TTCCACTGAT TCTACATTAT GGTGAGTTGT ATAATTATTT CATTATATAA TACAATGTAA TAATAATAGA AACACAGTGC ACAACAAATG TAATGTGCTT GAATCATCCC CAAACCATCC CAGTCCACGG TCTTCCACAT TTTGTCTTTT CACAAAATTG TCTTCCACAA AACTGGTCCC TGGTGCCAAA AAGGCTTGGG ACCACTGCTT TAAAGCCTTT GCATAGTGCT TAGAATTGAG GGGGAAAAAA AAAACAAAAA CAATGTAGCT AGTTGCTACA ATCACTATAT TGGTGAGTTT CAAAAGGAAA AGAATTCTGT CCCATTTATG CTTGAGCCTT GAGTTGCTAA CCAAGCCTGA CACAAAATTA CTGTTGAAGG GATGTGTGAG TCCTAATTGA AATGAGGCCT CTTAAGGGAA TTGTGGACCA AACCCCAAGC AGGCAGAAAG CCGTATCTTA ATTATTGCAA GTATTTCAGG CAAGGTGTGG ATGGCCATTT GAATTCAAGC AGACTAGGAC CTGGGATGAG AAAGAAGGTG TGTACGTGAC TTGATCTTTG AACTTTAGCT CACCATCTGG AAGAAGGCTG AGTATTCTCT GCACTCACAT AGTAGCTAAT GCCTACTCCC CAGCCACCCA CAATTCTTTC TGTAGGAAGG CTCGCTAGAA TACTTTGTGA TATTGGATAT TAGTTCCATA TTCTACTGTG TATCTTAGTT CAACCAAATT GTAATCATCT GATATTTATT TCTTTTAATA TAAATATAAG TATATTAAGT CTTGG

**Isoform 2**

ACAACATCGA CTTTGAAGTT GCACTGACCT GGCCAGCCCT GCCACTTACC AGGTTGGCTC TGTATGGCTA AGCGTTTTCT CCTAAAATCC CTTGAAAACT GTGAGAAGAC CATAAGAAGA TCATATCTTT AATTCTATTT CACAAGTCAC ACAATATTCC AATCAAATAC AGATGGTTGA GAAAAGTCAT CCATCTTCCC TCCCCACCCT CCCACAGCCC CTCAACCACT GCCCTGAAAC TTATATGCTG TTATCCGCAG CTCCATCTGG AGCATCACAG CTACTGTCAA CCCTGACGCT CTTTCTGAAA AAACACCGGA TGGACATCAG AACTATTTCT TTAAGGATGT TACTGAGCCA CACAGGAAAA CTTGCCTTAT GATTTTGAAT GCACGGATCT GATTTGACTA AACATGATAA CTAGAGAATC ACCCAATCTA CTCCCATTTT CAACTCTAAA TCATCAGAGT GTCTCAAATC CAAAGCACAC ACAGACCAGC CTGGCCAACA CGGTGAAACT CCACCCCTAC TAAAAGTATA AAAATTATCC AGGTGTGGTG GCGGGCGCCT GTAATCCAAG CTACTTGGGA GTCTGGAGGC AGGAGAATCC CTTGAACCTG GGAGATGGAG GTTGCAGTGA GCAGAGATCA CACCACCGCA CTCTAGCCTG GGCCACAAAT CAACAACAAC AACAACAACA AAAAACAAAG CGCACACAGA GACTGAGGTC CTCTTTGGCA TTGAGAAGAT GGCTATGCAA GTCCCAACTA GCAAGTGCAA ACTTCCCAGC TTCACTTCTG CCAGTGTCCC TTCACCCCTT CTCAACCCCA CTGGGAGGCA GGAGGGTGCT TGACAATAAC AGCCTTGGCA TCACTCTGCC AGGGTGTAAT AGGAACTGTT ACAATTCTGA GATTCTGTGT AAGCACTGGC CTTTCTGCCT AGAATGCCTT CTCCTCTCTT TTTTAACTGC ATGCTCCTAT TTATCTTTCA AAGCCCGGAA AAAATAACAC TGCACACGGG AAATGCTCCC TTCCTACTGC AGTCATTTAG ATGACTCTAT GCCATTCCAT TCATTTCTCT TTCCTACCAC AGAAGTGCTT TGAGATTTTG GAGTCAGACT GCTTGAACTT GAATCCTGGC CCTCTCATCA GAGACTTGAC TTATTTTAGG CAAGTTATAT AACCAATTTT ACCTCAGTTC CTTACCCATA AAATGGGTCT AATGAGAGTA CCTACCACAC AGAATTTTGA TGAAAACTGA ATGAGATGAA GGCCTTTAAG GCAGTGGTCC CCAACCCTGG GGACACAGAC AGGTACCATT TTGTGGCCTG TTAGGAACTG GGCCACACAG CAGGAGGTGA GCAGTGGGTG AGTGAGATCA GCGTTATTTA CAGCTGCTCC CCATTGCTCA CCTTACTGCC TGAGCTCCAC CTCCTGTCAG ATCAGCAGTG GCATTAAATT CTCATAGCAG CACAAACCCT GTCATGAACT GCACATGCGA GGGATCTAGG TTGTGCGCTC CTTATGAGAA TCTAATGCCT AATGACCTGT CACCGTCTCC CATCACCCCT AGATGGGAGT GTCTAGTTGC AGGAAACAAG CTCAGGGCTT CCACTGATTC TACATTATGG TGAGTTGTAT AATTATTTCA TTATATAATA CAATGTAATA ATAATAGAAA CACAGTGCAC AACAAATGTA ATGTGCTTGA ATCATCCCCA AACCATCCCA GTCCACGGTC TTCCACATTT TGTCTTTTCA CAAAATTGTC TTCCACAAAA CTGGTCCCTG GTGCCAAAAA GGCTTGGGAC CACTGCTTTA AAGCCTTTGC ATAGTGCTTA GAATTGAGGG GGAAAAAAAA AACAAAAACA ATGTAGCTAG TTGCTACAAT CACTATATTG GTGAGTTTCA AAAGGAAAAG AATTCTGTCC CATTTATGCT TGAGCCTTGA GTTGCTAACC AAGCCTGACA CAAAATTACT GTTGAAGGGA TGTGTGAGTC CTAATTGAAA TGAGGCCTCT TAAGGGAATT GTGGACCAAA CCCCAAGCAG GCAGAAAGCC GTATCTTAAT TATTGCAAGT ATTTCAGGCA AGGTGTGGAT GGCCATTTGA ATTCAAGCAG ACTAGGACCT GGGATGAGAA AGAAGGTGTG TACGTGACTT GATCTTTGAA CTTTAGCTCA CCATCTGGAA GAAGGCTGAG TATTCTCTGC ACTCACATAG TAGCTAATGC CTACTCCCCA GCCACCCACA ATTCTTTCTG TAGGAAGGCT CGCTAGAATA CTTTGTGATA TTGGATATTA GTTCCATATT CTACTGTGTA TCTTAGTTCA ACCAAATTGT AATCATCTGA TATTTATTTC TTTTAATATA AATATAAGTA TATTAAGTCT TAAAAAAAAA AAAAAAA
